# Supplementary material for: LINKIN, a new transmembrane protein necessary for cell adhesion
Source: eLife. 2014 Dec 1;3:e04449. doi: 10.7554/eLife.04449 (PMC4275582; doi:10.7554/eLife.04449)
Supplement: Supplementary file 2. — Top interactors of human LINKIN identified by mass spectrometry (>fivefold enriched over control). DOI: http://dx.doi.org/10.7554/eLife.04449.018 [file elife04449s002.docx]

| **Protein description** | **Human gene** | ***C. elegans* gene** | **RNAi** |
| --- | --- | --- | --- |
| Ig kappa chain V-I region Roy |  | no |  |
| Stromal cell-derived factor 2-like protein 1;PWP1-interacting protein 8 | *SDF2L1* | *R12E2.13* | I-1H06 |
| Calnexin;Major histocompatibility complex class I antigen-binding protein p88;p90;IP90 | *CANX* | *cnx-1/ crt-1* | III-5C10/ V-4H05 |
| DnaJ homolog subfamily B member 11 | *DNAJB11* | *dnj-20* | N/A |
| Mitochondrial glutamate carrier 1;Glutamate/H(+) symporter 1;Solute carrier family 25 member 22 | *SLC25A22* | *F20D1.9* | X-6P14 |
| Transcription factor Sp1;Sp1 transcription factor variant | *SP1* | no |  |
| T-cell immunomodulatory protein;Integrin-alpha FG-GAP repeat-containing protein 1 | *ITFG1* | *lnkn-1* |  |
| Mitochondrial 2-oxoglutarate/malate carrier protein;Solute carrier family 25 member 11 | *SLC25A11* | *misc-1* | II-1I19 |
| Solute carrier family 25 member 33;Bone marrow stromal cell mitochondrial carrier protein;HuBMSC-MCP;Protein PNC1;Solute carrier family 25 member 36 | *SLC25A33* | *T09F3.2* | II-7E02 |
| Lipolysis-stimulated lipoprotein receptor;Putative uncharacterized protein LSR;cDNA FLJ55699, highly similar to Homo sapiens liver-specific bHLH-Zip transcription factor (LISCH7), transcript variant 3, mRNA | *LSR* | no |  |
| Sigma 1-type opioid receptor;SR31747-binding protein;Aging-associated gene 8 protein | *OPRS1* | no |  |
| Vesicular integral-membrane protein VIP36;Lectin mannose-binding 2;Glycoprotein GP36b | *LMAN2* | *ile-2* | X-1K11 |
| Stromal cell-derived factor 2 | *SDF2* | *R12E2.13 (rep)* | I-1H06 |
| Probable glutathione peroxidase 8;Glutathione peroxidase | *GPX8* | *no* |  |
| Tubulin beta chain;Tubulin beta-5 chain | *TUBB* | *mec-7/ ben-1* | 10019-B11/ III-1F10 |
| Uncharacterized protein C7orf44 | *C7orf44* | no |  |
| ADP/ATP translocase 2 | *SLC25A5* | *ant-1.3* | IV-5C19 |
| Dolichyl-phosphate beta-glucosyltransferase;Asparagine-linked glycosylation protein 5 | *ALG5* | *H43I07.3* | N/A |
| RuvB-like 1;49 kDa TATA box-binding protein-interacting protein;TIP49a;Pontin 52;Nuclear matrix protein 238;54 kDa erythrocyte cytosolic protein;TIP60-associated protein 54-alpha;INO80 complex subunit H | *RUVBL1* | *ruvb-1* | V-6P06 |
| Phosphate carrier protein, mitochondrial | *SLC25A3* | *C33F10.12/ F01G4.6* | II-4E05/ IV-5N16 |
| Neutral alpha-glucosidase AB;Glucosidase II subunit alpha | *GANAB* | *aagr-3* | N/A |
| DnaJ homolog subfamily A member 1;Heat shock 40 kDa protein 4;DnaJ protein homolog 2;HDJ-2;HSJ-2;HSDJ;HDJ2 protein | *DNAJA1* | *dnj-12* | I-7I02 |
| Tubulin alpha-1C chain;Tubulin alpha-6 chain | *TUBA1C* | *tba-9* | X-2K23 |
| V-type proton ATPase subunit S1 | *ATP6AP1* | no |  |
| Tubulin beta-2C chain;Tubulin beta-2 chain;Tubulin beta 2C;cDNA FLJ53341, highly similar to Tubulin beta-4 chain | *TUBB2C* | *tbb-4* | X-4P09 |
| Desmoglein-2;HDGC | *DSG2* | no |  |
| Heat shock 70 kDa protein 13;Stress 70 protein chaperone microsome-associated 60 kDa protein;Microsomal stress 70 protein ATPase core | *HSPA13* | *stc-1* | II-6A08 |
| Tubulin beta-2B chain;Tubulin beta-2A chain | *TUBB2B* | *mec-7 (rep)* | 10019-B11 |
| Transcription factor RelB | *RELB* | no |  |
| Transmembrane protein 59;Liver membrane-bound protein;Transmembrane protein 59, isoform CRA_a | *TMEM59* | no |  |
| MHC class I antigen;HLA class I histocompatibility antigen, A-2 alpha chain | *HLA-A* | no |  |
| Zinc finger protein 470;Chondrogenesis zinc finger protein 1 | *ZNF470* | no |  |
| DnaJ homolog subfamily A member 2;HIRA-interacting protein 4;Cell cycle progression restoration gene 3 protein;Dnj3;Renal carcinoma antigen NY-REN-14 | *DNAJA2* | *dnj-19* | V-4C10 |
| ADP/ATP translocase 3;Adenine nucleotide translocator 2;ANT 3 | *SLC25A6* | *ant-1.3 (rep)* | IV-5C19 |
| Dynein, light chain, roadblock-type 1 | *DYNLRB1* | *dyrb-1* | II-6F21 |
| Tricarboxylate transport protein, mitochondrial | *SLC25A1* | *K11H3.3* | III-5E06 |
| RuvB-like 2;48 kDa TATA box-binding protein-interacting protein;TIP49b;Repressing pontin 52;51 kDa erythrocyte cytosolic protein;TIP60-associated protein 54-beta;INO80 complex subunit J | *RUVBL2* | *ruvb-2* | IV-3M02 |
| VIP36-like protein;Lectin mannose-binding 2-like | *LMAN2L* | *ile-2 (rep)* | X-1K11 |
| ATP synthase subunit gamma, mitochondrial;F-ATPase gamma subunit | *ATP5C1* | *Y69A2AR.18* | N/A |
| Occludin | *OCLN* | no |  |
| NADH dehydrogenase [ubiquinone] 1 alpha subcomplex subunit 4;NADH-ubiquinone oxidoreductase MLRQ subunit | *NDUFA4* | no |  |
| Type-1 angiotensin II receptor-associated protein;AT1 receptor-associated protein | *AGTRAP* | no |  |
| E3 ubiquitin-protein ligase RNF138;RING finger protein 138;Nemo-like kinase-associated RING finger protein | *RNF138* | no |  |
| Mitochondrial import inner membrane translocase subunit TIM50 | *TIMM50* | *scpl-4* | 11070-E9 |
| Transmembrane emp24 domain-containing protein 10 | *TMED10* | *F47G9.1* | 10001-A10 |
| Cdc42 effector protein 1;Binder of Rho GTPases 5;Serum protein MSE55 | *CDC42EP1* | no |  |
| Estradiol 17-beta-dehydrogenase 12;17-beta-hydroxysteroid dehydrogenase 12;3-ketoacyl-CoA reductase | *HSD17B12* | *let-767* | III-3I14 |
| Alpha-1,6-mannosyl-glycoprotein 2-beta-N-acetylglucosaminyltransferase | *MGAT2* | *gly-20* | V-7N19 |
| Signal peptidase complex subunit 3;Microsomal signal peptidase 23 kDa subunit;SPase 22 kDa subunit;SPC22/23 | *SPCS3* | *K12H4.4* | III-4A24 |
| 78 kDa glucose-regulated protein;GRP 78;Heat shock 70 kDa protein 5 | *HSPA5* | *hsp-3/4* | X-3G06 |
| Immunoglobulin heavy constant gamma 1 | *IGHG1* | no |  |
| Fatty acyl-CoA reductase 1 | *FAR1* | *Y71H10A.2* | X-2C13 |
| Isocitrate dehydrogenase [NADP], mitochondrial;Oxalosuccinate decarboxylase;NADP(+)-specific ICDH;IDP;ICD-M | *IDH2* | *idh-2* | X-5K18 |
| Beta-catenin | *CTNNB1* | *hmp-2* | I-7E20 |
| Chaperonin containing T-complex protein 1 subunit gamma | *CCT3* | *cct-3* | N/A |
| Phosphatidylserine decarboxylase proenzyme | *PISD* | *psd-1* | III-3L04 |
| Up-regulated during skeletal muscle growth protein 5 | *USMG5* | no |  |
| Elongation factor 1-alpha 1 | *EEF1A1* | *eft-3/ R03G5.1* | III-3N15 |
| Sideroflexin-4;Breast cancer resistance marker 1 | *SFXN4* | no |  |
| Transcriptional repressor protein YY1;Yin and yang 1;Delta transcription factor;NF-E1;INO80 complex subunit S | *YY1* | no |  |
| Calcium-binding mitochondrial carrier protein Aralar2;Mitochondrial aspartate glutamate carrier 2; Citrin | *SLC25A13* | *K02F3.2* | III-1C12 |
| Syntaxin 12 | *STX12* | *syn-13* | 10037-H11 |
| 4F2 cell-surface antigen heavy chain;Lymphocyte activation antigen 4F2 large subunit;4F2 heavy chain antigen | *SLC3A2* | *atgp-2, atgp-1* | II-9A08/ IV-8A02 |
| UDP-glucose:glycoprotein glucosyltransferase 1 | *UGCGL1* | *F48E3.3* | X-3L14 |
| N-acetyllactosaminide beta-1,3-N-acetylglucosaminyltransferase | *B3GNT1* | no |  |
| Dephospho-CoA kinase domain-containing protein | *DCAKD* | *T05G5.5* | 10056-B8 |
| Makorin-2;RING finger protein 62 | *MKRN2* | *Y55F3AM.6* | 11049-B5 |
| Nuclear pore membrane glycoprotein 210 | *NUP210* | *npp-12* | I-3G15 |
| Transferrin receptor protein 1 | *TFRC* | no |  |
