# Supplementary material for: LINKIN, a new transmembrane protein necessary for cell adhesion
Source: eLife. 2014 Dec 1;3:e04449. doi: 10.7554/eLife.04449 (PMC4275582; doi:10.7554/eLife.04449)
Supplement: Supplementary file 3. — lnkn-1 constructs used for rescuing lnkn-1 mutant. DOI: http://dx.doi.org/10.7554/eLife.04449.019 [file elife04449s003.docx]

| construct |  | primers | Restrict. sites used |
| --- | --- | --- | --- |
| Plasmid 1  p*lnkn-1* | Fire vector with a *lnkn-1* promoter insert. 4.1kb of lnkn-1 5’region was amplified by PCR from genomic DNA and inserted into pPD49.27 (Fire vector). | GGGTCGTGACAAAGTCAAAAACAT and CGCGGATCCAGTACCTGAACGTTGTTTTGTTTGAAG. | PstI and BamHI |
| Plasmid 2  p*lnkn-1*::*lnkn-1*(cDNA) | *lnkn-1* was amplified from cDNA and inserted into plasmid 1. | CGGGGTACCATGAAAAAAATATTACCGATCATATGGC and CGGGGTACCTACATGGCATCGAAATGGAATCG | KpnI |
| p*lnkn-1*::*lnkn-1* (signal seq. + ECD) | *lnkn-1* signal sequence and extracellular domain was amplified from cDNA and inserted into plasmid 1. | CGGGGTACCATGAAAAAAATATTACCGATCATATGGC and  CGGGGTACCCTAAGCTGATGGTGTGACGTAGA | KpnI |
| p*lnkn-1*::*lnkn-1* (signal seq.+ ECD+T) | *lnkn-1* signal sequence, extracellular and transmembrane domain was amplified from cDNA and inserted into plasmid 1. | CGGGGTACCATGAAAAAAATATTACCGATCATATGGC and CGGGGTACCCTATAAGAATACGACAACCATTAGAAGC | KpnI |
| p*lnkn-1*::*lnkn-1*(signal seq. + T + ICD) | *lnkn-1* transmembrane and intracellular domain, and unc-54 3’UTR was amplified from plasmid 2 and inserted into plasmid 1. XhoI cuts an internal site immediately after the signal sequence. | cgctcgagCTTATTGTTCAGAGTCTTGCCGTC and ATCACCGAAACGCGCGAGACGAAA | XhoI and ApaI |
| p*lnkn-1*::*lnkn-1* (ICD) | *lnkn-1* intracellular domain and unc-54 3’UTR was amplified from plasmid 2 and inserted into plasmid 1. | cggGGTACCATGCATTATCGAGAGAAAAAGGAAGAT and ATCACCGAAACGCGCGAGACGAAA | KpnI and ApaI |
| p*lnkn-1*::*lnkn-1*::YFP | Generated by PCR fusion. *lnkn-1* promoter and gene was amplified from plasmid 2. YFP and unc-54 3’UTR was amplified from L4817 (Fire vector) and fused by PCR. |  |  |
| p*lnkn-1*:: *YFP*::*lnkn-1* | YFP was amplified from L4817 (Fire vector) and inserted into plasmid 2. XhoI cuts a *lnkn-1* internal site immediately after the signal sequence. | GCAAAAACCAGAATATCAGTATTCC and CTCTTCAAACAAAACAACGTTCAGG | XhoI |

Table S3. *lnkn-1* constructs used for rescuing *lnkn-1* mutant
